# Construction of Physical Education Quality Evaluation Index and Analysis with Wearable Device

**DOI:** 10.1155/2022/1190394

**Published:** 2022-05-26

**Authors:** Lei Fang

**Affiliations:** Jilin Agricultural Science and Technology University, Jilin 132101, China

## Abstract

As an important part of the education system, college physical education directly affects the comprehensive development of college students' physical and mental quality. It is necessary to build a scientific and efficient evaluation index of college physical education teaching quality. Physical fitness monitoring is an important indicator for the quality evaluation of physical education. However, how to achieve lightweight, portable, and high-accuracy quantitative physical fitness monitoring is currently a major challenge. In order to solve the above challenges, this paper proposes a method of constructing a physical education quality evaluation index based on wearable devices. The wearable device collects human ECG signals, calculates the exercise intensity of participating students, and realizes quantitative evaluation of the quality of physical education teaching. Aiming at the problems of complex equipment and low accuracy of the existing exercise intensity detection methods, this paper proposes an ECG signal wave group detection algorithm based on a one-dimensional convolutional neural network (1D-CNN) to obtain the heart rate variability signal more accurately. After obtaining the ECG feature vector, the SVM classifier is used to predict the exercise intensity. In order to verify the effectiveness of the method in this paper, the real data collected from students of one university and a public available dataset are selected for experiments. The experimental results show that the method proposed in this paper achieves a good performance.

## 1. Introduction

Physical education [1] plays an important educational role in the education and training goals of colleges and universities. As an indispensable and important educational link, the quality of physical education teaching directly affects the training of talents' comprehensive quality. The quality evaluation system should be conducive to the healthy and harmonious development of college students. Physical fitness monitoring is an important indicator of physical education quality evaluation [2]. Lightweight, portable, and highly accurate quantitative physical fitness monitoring is very important for building a scientific physical education quality evaluation system. In the process of sports training, it is an important part of scientific physical education to effectively monitor the physiological parameters of athletes and objectively assess the physical function state of athletes: excessive exercise will cause loss of organs and internal organs, causing irreversible damage to athletes. Insufficient exercise will not achieve the effect of exercise, making sports inefficient.

Heart rate [3] is a technical term used to describe the cardiac cycle and also refers to the number of times the heart can contract per minute during exercise. Heart rate is one of the easiest indicators to measure the cardiovascular system. It can reflect not only cardiovascular function but also the degree of energy saving and recovery of the body and has high reliability. Heart rate variability is an index to evaluate the function of the autonomic nervous system, which can reflect the activity and coordination of sympathetic and parasympathetic nerves. With the deepening of research, it has been gradually introduced into the monitoring indicators of physical fitness in the process of physical training. Changes in heart rate are the most reliable indicators for monitoring exercise load.

Easy to wear, scientific records, etc. are the issues that should be paid attention to in physical exercise monitoring. With the development of electronic information technology, the continuous reduction of chip size, the diversification of sensor data collection, and the continuous improvement of computing speed, wearable exercise monitoring equipment can well meet this challenge. This paper proposes a method for constructing a physical education quality evaluation index based on wearable devices. The wearable device collects human ECG signals, sends them to the computer through Bluetooth for processing, and calculates the exercise intensity of the participating students. It can help physical education teachers to grasp the students' physical fitness, functional state, and fatigue. Thus, this collected information can favor scientific control and adjustment of curriculum development, to achieve quantitative assessment of the quality of physical education. The ECG signal will inevitably be interfered by various noise signals during the acquisition process, which will seriously affect the detection efficiency in the automated testing process. In addition, the accuracy of R-wave detection is the key problem for heart rate variability signal extraction that should be solved first. In order to solve this problem, this paper proposes an ECG signal wave group detection algorithm based on a one-dimensional convolutional neural network to obtain the heart rate variability signal more accurately. After obtaining the ECG feature vector, the SVM classifier is used to predict the exercise intensity.

The main contributions of our paper can be concluded as follows:

1. A novel physical education quality evaluation method based on wearable devices is proposed. By collecting ECG signals and monitoring students' physical fitness, the quantitative evaluation of physical education quality is realized.
2. An ECG signal processing method based on a one-dimensional convolutional neural network is proposed.
3. A exercise intensity prediction algorithm based on an SVM classifier is proposed to monitor the physical fitness of participants.
4. The experimental evaluation is launched on the data collected from students of one university and public available dataset. The results show that the proposed method in this paper has good performance.

The following paper is organized as follows: Section 2 details the related work.  Section 3 provides the detail description about our proposed method.  Section 4 evaluates our method based on data collected from the participants as well as data from the public available datasets. Finally, Section 5 concludes our paper.

## 2. Related Work

At present, China is actively exploring the combination of smart wearable devices and school sports work. With the advanced technical support, portability, and interactivity of smart wearable devices, it is applied to school sports work to serve school sports. The study [4] pointed out that the current physical condition of students is on the decline. By developing a wearable intelligent human body exercise intensity monitoring system, the physical exercise process of students can be monitored so that the physical exercise of students is in a reasonable intensity process [5]. Besides, it can effectively improve the sports efficiency and improve the physical health of students. The American College of Sports Medicine conducts an annual survey of 2,800 fitness experts in the United States, and the results show that the use of smart wearable devices has become the first of seven fitness trends. Accurate data, considerate services, and portability provide strong support for the popularization of smart wearable devices. With the improvement of people's living standards, more and more attention is paid to their physical health. Wearable devices provide users with all-weather services, which can motivate users to choose a healthier lifestyle.

Heart rate is an important indicator for health assessment and disease diagnosis, which can be continuously and dynamically monitored by wearable physiological sensing technology [6]. Heart rate can be used as an objective evaluation index of the physiological load of human exercise, and it is of great significance in preventing the injury of high-intensity physical training and helping to formulate a personalized fitness plan [7]. Since most algorithms calculate heart rate by extracting the time interval between adjacent R-wave peaks, accurate heart rate detection relies on accurate localization of the QRS complex [8].

Deep learning is a branch of artificial neural networks in machine learning. It allows computers to learn data representations with multiple levels of abstraction using computational models with multiple processing layers [9]. It has been used in many disciplines such as computer vision, speech recognition, natural language processing, and bioinformatics [10, 11]. More and more deep learning-based methods are used to study the problem of arrhythmia diagnosis. Mathews et al. [12] proposed a deep learning-based arrhythmia classification algorithm, using single-lead ECG signals with a sampling rate of 114 Hz for arrhythmia classification. Pławiak and Acharya [13] used 10s ECG signal segments to enhance features through power density estimation and proposed a novel 3-layer deep genetic classifier for arrhythmia classification. The study [14] proposed a data augmentation technique using Generative Adversarial Networks (GAN) to balance the dataset and effectively improve the performance of the same model for ECG classification. Li et al. [15] proposed an intelligent recognition algorithm for ECG arrhythmia signals based on wavelet adaptive threshold filtering and deep residual convolutional neural network (DR-CNN), which effectively improved the overall recognition accuracy rate, sensitivity, and specificity of ECG arrhythmia signals.

Šarlija et al. [16] proposed a QRS complex detection algorithm based on a one-dimensional convolutional neural network (1D-CNN). Xiang et al. [17] applied the attention-based two-level 1D-CNN method for QRS complex detection. First, the differential signal and the average differential signal of the ECG signal were obtained through data preprocessing, and then the different signals were sent to the object level. CNN and part-level CNN are used to extract features of different granularities of ECG signals. Finally, the morphological features extracted by object-level CNN and part-level CNN are fused and provided to multilayer perceptron for QRS complex detection. However, the above algorithm needs to perform multiple preprocessing operations on the signal, which is equivalent to filtering the signal, which will filter out useful information in the ECG signal and increase the computational complexity of the algorithm.

## 3. Our Method

**Figure 1:**
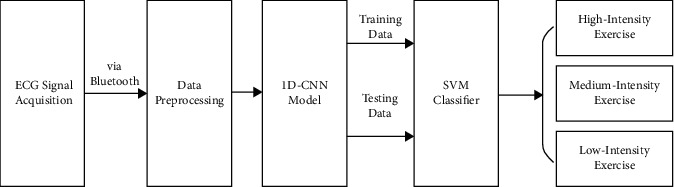
Overall architecture of our method.

The overall architecture of the method proposed in this paper is shown in Figure 1. First, the ECG signals of the students participating in the course are collected through the wearable device. Then, the wearable device transmits the data to the computer through Bluetooth. Next, the collected data are preprocessed. In this phase, a one-dimensional convolutional neural network (1D-CNN) model is exploited to analyze the data and extract features of QRS complex. Finally, the ECG feature vector obtained in the previous stage is input into the SVM classifier as training data A classifier with three levels of exercise intensity is obtained. During the running phase, the test data are fed into the trained SVM classifier to detect the exercise intensity of the participating students. It can be helpful for physical education teachers to grasp the physical function state and fatigue of students. Thus, the course can be controlled and adjusted scientifically and quantitatively to improve the quality of physical education teaching.

### 3.1. ECG Signal Acquisition Based on Wearable Devices

**Figure 2:**
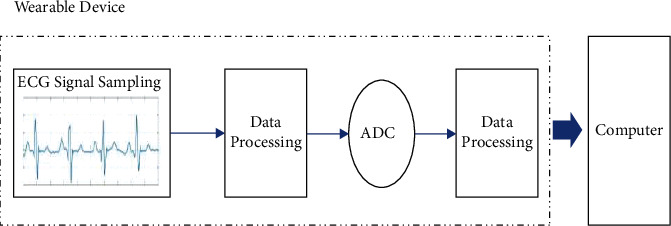
Workflow diagram of the wearable device.

In this paper, the IREALCARE2.0 flexible remote ECG patch is used as a wearable device to monitor heart conditions and collect ECG signals. It has the characteristics of small size, light weight, and accurate and reliable measurement data. Its core electrical signal measurement chip is ADS1291, which is a low power 2-channel 24 bit analog front end for biopotential measurement. After the signal acquisition is completed, through filtering, amplification, and other operations, the signal is input to the ADC, converted into a digital signal, and finally transmitted to the back-end computer through Bluetooth for data analysis and processing. The workflow diagram of the wearable device can be seen in Figure 2.

### 3.2. 1D-CNN Based ECG Feature Vector Output Algorithm

In this paper, the QRS complex detection algorithm based on a one-dimensional convolutional neural network (1D-CNN) is exploited to analyze and process the data transmitted by the wearable device and to obtain the ECG feature vector. The specific process is as follows.

#### 3.2.1. Data Preprocessing

There are three main types of ECG signal noise, namely EMG interference, power frequency interference, and baseline drift [18]. For these three kinds of noise signals, different filters are used to filter out these three kinds of noise signals, respectively. The frequency of the ECG signal is between 0.01 Hz and 100 Hz [19], generally concentrated in 0.25–40 Hz. However, the frequency distribution of EMG signals is wide, usually between 40 Hz and tens of thousands of Hz. For example, the EMG noise of a single fiber is between 500 Hz and 10000 Hz [20]. Relatively speaking, the ECG signal is located in the low-frequency band, so a low-pass filter is used to filter out the EMG interference signal. The power frequency interference usually includes 50 Hz or 60 Hz sine wave and its harmonic signal [21]. Therefore, the 50/60 Hz notch filter is designed by the window function method; that is, a low-pass filter is used to superimpose a high-pass filter to filter out. The baseline drift noise is generally between 0.05 Hz and 2.0 Hz, which is close to the frequency of the ECG signal [22], and IIR digital filter is used to correct it as much as possible.

#### 3.2.2. Residual Connection

CNN has the characteristics of local connection and weight sharing [23], parameter sharing can effectively reduce the problem of overfitting, and sparse connection can allow the network to learn local features. One-dimensional convolutional neural network [24] is a convolutional neural network that uses one-dimensional convolution to extract features from one-dimensional time series sequences, which can ensure the extraction of local features without losing time series features.

Existing studies have found that the deeper the network is built, the easier it is to have gradient disappearance and network overfitting. Residual connections can effectively solve the problems of network degradation and gradient disappearance. The idea of residual connection is derived from the gating idea of LSTM, which transforms the input nonlinearly and then performs linear superposition output with the input.

One layer of the network can usually be regarded as *y* = *H* (*x*), and the output of the residual block of the residual network can be expressed as *H* (*x*) = *x* + *F* (*x*), that is to say, the residual is the predicted value. The difference between *H* (*x*) and the observed value *y* = *x*, that is, *F* (*x*) = *H* (*x*) − *x*. The learning objective of the residual network is changed from learning *H* (*x*) to learning residual *F* (*x*). The network only needs to learn the difference between input and output, thereby reducing the difficulty of learning. The output formula of the residual block is as follows:

(1) $$\begin{array}{c} x_{l+1}=x_{l}+F\left(x_{l},w_{l}\right). \end{array}$$

In the formula, *x*_*l*+1_ is the output of the *l* + 1th layer; *x*_*l*_ is the input of the *l*_th_ layer; and *F*(*x*_*l*_, *w*_*l*_) is the residual of the *l*_th_ layer. Skip connections can quickly feed back to another layer or even deeper layers after a network layer is activated, thereby avoiding the loss and loss of information in traditional convolutional layers and fully connected layers during information transfer. Using skip connections can build a deep network to train a deeper network, which can ensure that the network parameters remain unchanged and the amount of computation does not increase, while ensuring that the network has sufficient capacity to process more complex data.

#### 3.2.3. 1D-CNN Model

**Figure 3:**
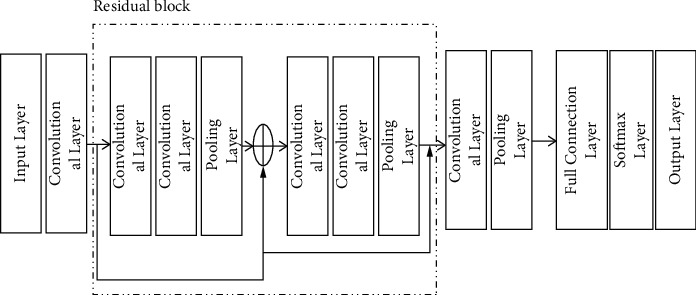
Overall architecture of 1D-CNN model.

The common two-dimensional convolutional neural network is often used for image classification. The ECG signal to be processed in this paper is a one-dimensional discrete sequence. So, the two-dimensional convolutional neural network is first modified to a one-dimensional convolutional neural network [25] to be suitable for ECG signal feature extraction. The one-dimensional convolutional neural network structure used in this paper is shown in Figure 3.

It consists of 1 input layer, 6 convolutional layers, 3 pooling layers, 1 fully connected layer, a Softmax layer, and an output layer. First convolve the input once and then divide the convolution output into two parts: one part is saved as identity and the other part continues to be input to the residual block part. The result of 2 convolutions is added to the saved identity to obtain the residual output. Use 2 residual blocks and perform two skip connections to form the entire residual connection part. The obtained results go through a convolutional layer and a pooling layer to finally get the extracted features. Finally, the output of the pooling layer is passed through a fully connected layer and a Softmax layer to obtain the final output result.

The convolution layer performs a convolution operation between the feature vector of the upper layer and the convolution kernel of the current layer. Relu is used as the activation function to enhance the characteristics of the original signal and reduce noise. The parameters of the convolution layer include the length of the convolution kernel, the sliding step length, and padding; the three together determine the length of the output feature vector of the convolutional layer. The length of the convolution kernel can be specified as any value smaller than the input feature vector. The larger the convolution kernel, the more complex the features that can be extracted. The sliding step size is the sliding length of the convolution kernel in the horizontal direction of the feature vector each time. For example, if the sliding step size is 2, the convolution kernel moves once every other point. As the convolutional layers are stacked, the length of the feature vector gradually decreases. For this reason, the purpose of padding is to artificially increase the length of the feature vector to offset the reduction in the length of the feature vector in the calculation. Common padding methods are Same and Valid. The padding method used in this paper is Valid, which is nonpadding, and P is 0. The formula for calculating the length *X* of the output feature vector after each convolutional layer is as follows:

(2) $$\begin{array}{c} X=\frac{W-F+2P}{S}+1. \end{array}$$

In the formula, *W* is the length of the input data, *F* is the length of the convolution kernel, the value of *P* is determined according to the type of all 0 padding, and *S* is the sliding step size of the convolution kernel.

Pooling, also known as downsampling, does not change the number of input feature vectors, but only changes the length of each feature vector, mainly to reduce model complexity and reduce overfitting. The most commonly used pooling methods are maximum pooling and average pooling. In this paper, maximum pooling is used to reduce the dimension of ECG data, compress features, and extract effective features. In the feature vector area covered by the pooling kernel, the maximum value is selected to replace all values in the current area [26]. The formula for calculating the length *Y* of the output feature vector after each pooling layer is as follows:

(3) $$\begin{array}{c} Y=\frac{W-F}{S}+1, \end{array}$$

where *W* is the length of the input data, *F* is the length of the pooling kernel, and *S* is the sliding step size of the pooling kernel. Finally, the output of the fully connected layer is passed through Softmax to obtain the probability value to calculate the classification result.

### 3.3. SVM Classifier-Based Exercise Intensity Classification Method

The SVM classifier [27] is adopted as the classification and discrimination mechanism of different exercise intensities. In the previous stage, the 1D-CNN model is used to extract the ECG feature vectors. Next, the ECG feature vectors are fed into the SVM classifier to train the classification model to achieve the goal of classification of the exercise intensity.

In this paper, exercise intensity is divided into three levels, namely low intensity, moderate intensity, and high intensity. According to the sports intensity classification standard issued by the Institute of Sports Science of the General Administration of Sport of China (Table 1), the metabolic equivalent of task (MET) value is 3 for low-intensity exercise, 3 to 6 for moderate-intensity exercise, and greater than 6 for high-intensity exercise. For example, the MET value obtained from ActiGraph wGT3X-BT should be moderate in intensity after comparing with Table 1. At the same time, the relevant features of the ECG signal are calculated and input into the SVM as a feature vector to judge the motion level.

SVM multiclassification construction [28, 29]: Since the SVM pair initially appeared as a binary classifier, the multiclassification effect is not good. In this paper, the voting method of pairwise classification is adopted, and the SVM classifier is changed to a multiclass classifier. After many experiments, the Radial Basis Function (RBF) is selected as the kernel function of SVM classification, the *γ* value is set to 1, and the penalty factor coefficient is 2.

## 4. Experimental Evaluation

### 4.1. Experimental Settings

Considering that the physical education quality evaluation method proposed in this paper first uses the 1D-CNN model to calculate the collected ECG data and then uses the SVM classifier to evaluate the exercise intensity, this paper conducts experimental evaluations on these two parts, respectively. In this section, we first show the experimental results of the proposed method on exercise intensity evaluation under different sports. The performance of the 1D-CNN model is then evaluated using public datasets. The metrics to evaluate our method are listed as follows, which are Accuracy, Precision, Recall, and *F*-Score, respectively:

(4) $$\begin{array}{c} \text{Accurcy}=\frac{TP+TN}{TP+TN+FP+FN},\\ \text{Precision}=\frac{TP}{TP+FP},\\ \text{Recall}=\frac{TP}{TP+FN},\\ \text{F}-\text{score}=\left(1+\beta^{2}\right)\frac{\text{Precision}\cdot \text{Recall}}{\beta^{2}\left(\text{Precision}+\text{Recall}\right)}. \end{array}$$

Among them, *β* is used to balance the weights of Precision and Recall in the *F*-score calculation. In general, *β* takes 1. TP is the number of positive samples predicted as positive samples, TN is the number of negative samples predicted as negative samples, FP is the number of negative samples predicted as positive samples, and FN is the number of positive samples predicted as negative samples.

### 4.2. Evaluation on Exercise Intensity under Different Exercises

In our work, 32 college students aged 22 to 28 from a university were selected, including 22 boys and 10 girls. The 32 people were evenly divided into 2 groups according to age, gender, and physical fitness, and each group had 16 people, namely Group 1 and Group 2. The data of Group 1 were used as training data, and the data of Group 2 were used as test data. All students were asked to carry out 4 sports training of uniform running, table tennis, badminton, and basketball.

The tester wears the ActiGraph wGT3X-BT device [30] and the wearable device on the front chest at the same time. ActiGraph wGT3X-BT provides metabolic equivalent of task values and collects ECG signals through wearable devices. ActiGraph wGT3X-BT is a human motion energy consumption detector, which provides MET data, and MET is the metabolic equivalent of task.

**Figure 4:**
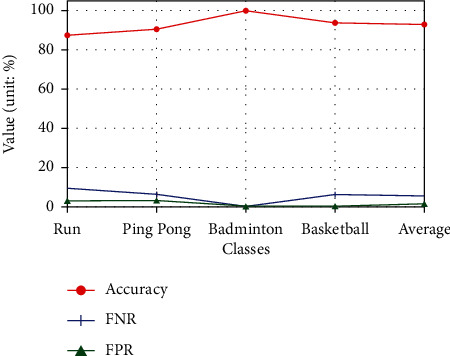
The validity evaluation of exercise intensity with different sports.

The results are shown in Figure 4. The experimental results of different sports are different. The accuracy rate of these four kinds of sports (the order is: run, ping pong, badminton, and basketball) are 87.5%, 90.625%, 100%, and 93.75%, respectively. The average accuracy among these results is 92.97%. The false-negative rates are 9.375%, 6.25%, 0%, and 6.25%, respectively, while the false-positive rates are 3.125%, 3.125%, 0%, and 0%, respectively. The average false-negative rate and the average false-positive rate are 5.47% and 1.56%.

Among the results we can see that, badminton has the highest accuracy rate, which can accurately evaluate the exercise intensity of all participating objects. Basketball is the next most accurate, then ping pong. The recognition accuracy of uniform running is the lowest. This paper believes that the exercise intensity during uniform running is obviously less fluctuating than other sports, which may be the reason for the low recognition rate. This also shows that the method proposed in this paper is more sensitive to high-intensity motion detection. In general, the average accuracy of the method proposed in this paper reaches 92.97%, which can meet the needs of daily courses considering the wearable device scene. Therefore, the algorithm proposed in this paper can carry out effective exercise intensity detection, which can help physical education teachers to grasp the physical function state and fatigue status of students. In addition, it can also control and adjust the course scientifically, and realize quantitative evaluation of the quality of physical education teaching.

### 4.3. Evaluation on 1D-CNN-Based ECG Signal Processing Algorithm

#### 4.3.1. Datasets

To evaluate the effect of the 1D-CNN model exploited in our paper, the publicly available dataset MIT-BIH is used in this section. The MIT-BIH arrhythmia database includes 48 dual-channel Holter recordings, the first 23 records were extracted from routine outpatient data, and the remaining 25 records were due to the inclusion of some rare, complex, and difficult to identify chambers. The 48 dual-channel signal distributions are shown in Table 2. The normal QRS complex is usually prominent in the first channel, and the signal lead axis of the second channel is almost orthogonal to the average ECG axis. In order to accurately locate the position of the QRS complex, the first-channel signal is used in this paper. In the MIT-BIH arrhythmia database, 46 records were selected (102 and 104 records did not come from MLII leads, and this paper did not use them as a dataset) and divided them into a training set DS1 and a validation set DS2 in a cross-patient manner. Where DS1 = (101, 106, 108, 109, 112, 114, 115, 116, 118, 119, 122, 124, 201, 203, 205, 207, 208, 209, 215, 220, 223, 230), DS2 for the remaining 24 records [31].

#### 4.3.2. Evaluation on Varied Dataset Length

In this paper, the R wave peak and random non-R wave peak positions in the QRS complex are used as the center, and a fixed window is used to intercept the data to form ECG signal segments. According to the characteristics of ECG signals, this paper takes the R wave peak and random non-R wave peak positions provided by the MIT-BIH arrhythmia database as the center to take 50, 75, and 100 sampling points, respectively, to form 101, 151, and 201 sampling points length dataset.

**Figure 5:**
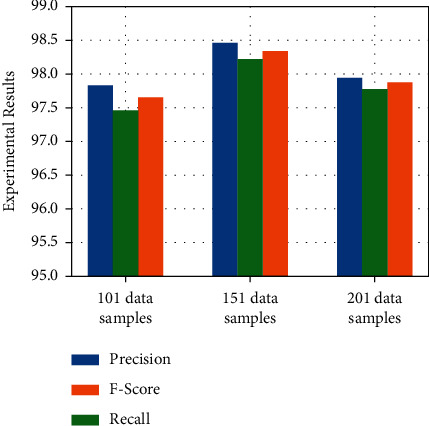
Experimental evaluation on varied dataset length.

The experimental results are shown in Figure 5. In general, different dataset lengths have achieved relatively good experimental results, and the minimum precision is over 97% among these three datasets. As can be seen from Figure 5, the experimental results of the dataset length of 151 sampling points are in three. Each index is better than other length datasets. The experimental results with a dataset length of 201 sampling points are slightly higher than those with a dataset length of 101 sampling points. Therefore, the length of the dataset is selected as 151 sampling points to achieve the best experimental results.

#### 4.3.3. Evaluation on the Relationship between the Number of Iterations and the Accuracy

**Figure 6:**
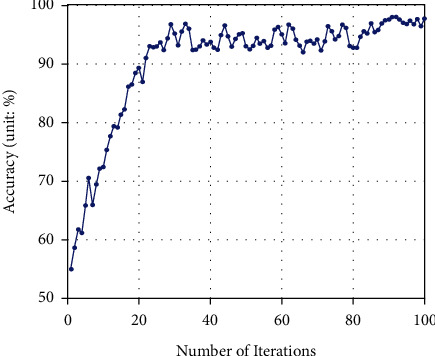
Evaluation on accuracy over iteration during training.

**Figure 7:**
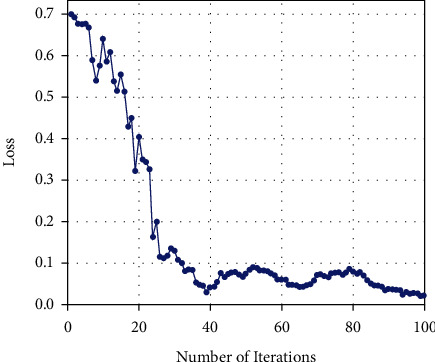
Evaluation on loss over iteration during training.

The number of iterations of CNN model training can have an impact on the experimental results. In this study, the cross-entropy function is used as the loss function, and the loss function is the optimization target of the model. The smaller the cross-entropy, the more accurate the prediction result. Set the number of training iterations to 100 and evaluate the change of the loss function and model accuracy with the number of iterations. As shown in Figure 6, as the number of iterations increases, the loss function of the model gradually decreases and tends to be stable, and the loss function finally converges to around 0.02. The changes in the accuracy of the model classification and recognition are shown in Figure 7. It can be seen that with the increase of the number of iterations, the classification accuracy of the model gradually increases and tends to be stable, and finally converges to about 98%.

## 5. Conclusion

Portable and quantifiable physical fitness monitoring is helpful to build a scientific evaluation system of physical education teaching quality in colleges and universities, which is of great benefit to the cultivation of talents' comprehensive quality and to the healthy and harmonious development of college students. Heart rate is an important indicator for health assessment and disease diagnosis. It can objectively evaluate the physiological load of human exercise and can be continuously and dynamically monitored by wearable physiological sensing technology. Aiming at the problems of low accuracy and inconvenience of existing methods, this paper proposes a method for constructing physical education quality evaluation indicators based on wearable devices. The wearable device collects ECG signal of the participant and computes the exercise intensity to achieve the goal of quantitative assessment of physical education teaching quality. In order to solve the problem of noise interference in the process of ECG signal extraction, which affects the detection efficiency, a one-dimensional convolutional neural network model is introduced in this paper. After the ECG feature vector is output, the SVM classifier is used to efficiently classify the exercise intensity of the participants. The experimental results show that the method proposed in this paper can achieve continuous and convenient physical monitoring, and at the same time can obtain good performance. This is of great significance for helping physical education teachers to grasp the physical state and fatigue of students, scientifically control and adjust curriculum development, and achieve a quantitative evaluation of physical education teaching quality.

## Figures and Tables

**Table 1:** The standards of exercise intensity.

Exercise intensity	Low intensity	Medium intensity	High intensity
MET value	≤3	3∼6	≥6

**Table 2:** Channel distribution of each record in the MIT-BIH arrhythmia database.

Channel1	Channel2	Record
MLII	V1	101 105 106 107 108 109 111 112 113 115 116 118 119 121 122 200 201 201 203 205 207 208 209 210 212 213 214 215 217 219 220 221 222 223 228 230 231 232 233 234
MLII	V2	103 117
MLII	V4	124
MLII	V5	100 123
V5	MLII	114
V5	V2	102 104

## Data Availability

All data used to support the findings of the study are included within the article.
